# Can Malaysian Young Adults Report Dietary Intake Using a Food Diary Mobile Application? A Pilot Study on Acceptability and Compliance

**DOI:** 10.3390/nu9010062

**Published:** 2017-01-13

**Authors:** Yoke San Chen, Jyh Eiin Wong, Ainaa Fatehah Ayob, Nor Effendy Othman, Bee Koon Poh

**Affiliations:** 1 Nutritional Sciences Programme, School of Healthcare Sciences, Faculty of Health Sciences, Universiti Kebangsaan Malaysia, 50300 Kuala Lumpur, Malaysia; sammcys@gmail.com (Y.S.C.); ainaafatehah@siswa.ukm.edu.my (A.F.A.); pbkoon@ukm.edu.my (B.K.P.); 2 Research Center for Software Technology and Management, Faculty of Information Science and Technology, Universiti Kebangsaan Malaysia, 43600 Bangi, Malaysia; effendy@ukm.edu.my

**Keywords:** dietary assessment, mobile food record, image-based assessment, acceptability, compliance

## Abstract

Mobile applications may improve dietary reporting among young adults due to their high accessibility and embedded camera function. This pilot study aimed to (i) evaluate users’ acceptability and compliance in reporting dietary intake using a newly developed food diary mobile application (food app); and (ii) identify issues and recommendations for improving dietary assessment using this food app via quantitative and qualitative protocols. Twenty-eight university students each used a food app for seven consecutive days and attended one of five focus group interviews. A 42% decrement in reporting compliance was observed throughout the seven-day recording period. An average of 5.9 recording days were reported and 4.8 occasions of meal data were uploaded each day. Based on questionnaires, high levels of agreement were reported in terms of perceived usefulness (69.3%), perceived ease of use (77.1%), attitude (73.6%), perceived enjoyment (62.6%), and smartphone experience (91.1%), but such agreement was not reported for intention to use (38.1%) and social influence (33.4%). Four major themes emerged from the focus group interviews, namely, (i) features; (ii) potential use; (iii) utility issues of the food app; and (iv) suggestions for improvements. While the food app was well-accepted by most of the young adults, the current prototype would benefit from incorporation of a barcode scanning function, customizable reminders, in-app tutorial, an entertainment component, and enhancement in overall appearance.

## 1. Introduction

Accurate dietary assessment is fundamental to assess nutritional status and establish diet-disease relationship [1,2,3]. Paper-based food record methods such as weighed and estimated food records are less memory-reliant than retrospective methods in providing quantitative information on food intake. However, such recording methods have their own limitations [4]. These prospective methods require users to record every detail of their food consumption and are thus burdensome and may lead to inaccurate reporting [5,6].

New dietary assessment approaches which employ digital data communication, such as electronic food diary in the form of a mobile application, may offer advantages over paper-based food records in assessing dietary intake [7]. Through wireless networks, mobile applications serve as platforms for data collection in different formats (images, texts, and video) [8] and therefore enable capturing of food intake in real-time and help reduce the burden of both the record keeper as well as the researcher [1]. Assessing dietary intake of young adults is challenging as they have high day-to-day variability in their daily diet [9] due to unstructured eating patterns and frequent outside-of-home meals [10,11]. Moreover, younger generations reported low compliance using paper-based dietary assessment but showed preference towards using technology to aid in their dietary assessment [12,13]. Therefore, employing a mobile application for dietary assessment in this age group is promising.

To improve dietary assessment using mobile applications, it is important to employ a tailored mobile application which fits into and is easily incorporated into the target groups’ lifestyle [14,15]. For this purpose, an image-based food diary mobile application (food app) prototype has been developed to enable electronic dietary assessment among Malaysians. Based on a server-client model, this food app allows users to upload images and text descriptions of their meal and leftovers to a central server for further nutrient analysis by a research team. Prior to validating this mobile application, acceptability evaluation is important to ensure that the entire design process works in an easy and understandable way, as well as to deliver an enjoyable experience to the users interacting with the tool. The level of compliance which indicates the usefulness of a tool [6] can be measured by examining the willingness of users to continue using a newly developed tool and their ability to follow the instructions or protocols for a certain period of time with ease. Therefore, the purpose of this pilot study is to examine the acceptability and compliance of young adults in recording dietary intake using this newly developed food app.

## 2. Materials and Methods

This study employed both quantitative and qualitative methods to examine the users’ acceptability and compliance towards a new food app. Study recruitment was conducted among university students of the Faculty of Health Sciences, Universiti Kebangsaan Malaysia (UKM), Kuala Lumpur through advertisements on social media channels (Facebook and WhatsApp groups) and word-of-mouth. Using purposive sampling, 28 participants aged 18 to 24 years who are English-literate and who each owned a smartphone with an Android operating system (4.0 and above) were recruited into this study. The study protocol was approved by the Research Ethics Committee of Universiti Kebangsaan Malaysia (NN-149-2014). All participants were given a briefing on the project and completed a written, informed consent form prior to participation.

### 2.1. Dietary Reporting Using the Food App

**Figure 1:**
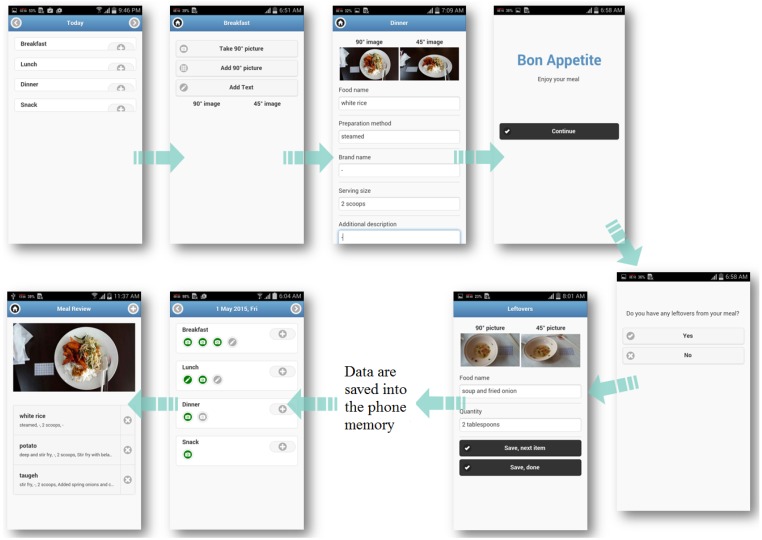
Illustration of food recording process using the food app.

A newly developed Android-based food app was installed on each participant’s smartphone. This food app was developed as a hybrid application through HTML5 and Javascript using PhoneGap (Adobe Systems Inc., San Jose, CA, USA). On the food log page, users could record their food intake according to eating occasions (breakfast, lunch, dinner, and snacks) either by using the Camera function (to capture food images), Text function (to input text information), or both functions (see Figure 1).

In groups of four to five members, participants were provided with approximately 30 min of training and practice to ensure they understood and were familiar with the operation of the food app. A written manual on the operation of the food app was distributed to the participants for their reference. Participants were required to use the food app for seven consecutive days to report their food and beverage consumption.

Following a standardized protocol for dietary reporting, the participants were instructed to take two images, namely an aerial (90°) view and an angled (45°) view of their food before eating; and another two of any leftovers after eating. The two images were required for food identification and volume estimation [16]. Additionally, participants were instructed to place a fixed sized-card next to their food every time they take their food images. This customized card was made using a standard graph paper with printed black lines and numbers to mark each 1-centimeter grid (8 cm × 5 cm). It was laminated to be similar in size to a standard credit card (8.5 cm × 5.4 cm) and served as a fiducial marker for volume estimation in this study (Note that a fiducial marker is seen on the left of a plate and right of a bowl in food images of Figure 1). Besides, participants were encouraged to provide text inputs about the food in five designated text boxes; namely food name, serving size, preparation method, brand name, and additional information (Figure 1). On occasions when it was not possible to take photographs, participants may use the Text function to provide textual information only. Data entered in the food app was stored into an SQLite database, which resides in the participant’s phone. When internet was available, these data were sent to and stored on a remote server (My SQL database) when the user synced the food app with the server. Each participant was given a small cash reimbursement to cover the cost of their mobile internet plan.

### 2.2. Focus Group Discussion

After completion of food records for seven days, participants were invited to attend one of the five focus group sessions to discuss issues and problems encountered throughout the food-recording period. Each focus group session was facilitated by a moderator and comprised five to seven participants who were grouped based on homogeneity according to experience in food recording using paper-based methods. Semi-structured questions were developed based on the Krueger and Casey model [17]. All interviews were audio-recorded using a voice recorder (Model ICD-UX543F, Sony, Japan) and subsequently transcribed verbatim. Transcripts were analyzed by a researcher (Y.S.C.) in a systematic manner which involved extensive initial reading of transcripts; indexing the themes within the transcripts; removing data from the transcripts; and transferring the data into relevant themes [18]. An independent researcher (A.F.A) reviewed all the data to confirm that similar quotes were clustered into categories to arrive at the same major themes.

### 2.3. Compliance and Acceptability Evaluation

All participants completed a paper-based questionnaire to rate their acceptance of the food app based on the Technology Acceptance Model (TAM) [19]. This questionnaire contained 29 items validated in previous studies [20,21,22,23,24] and modified to fit the specific context of the food app. Using five-point Likert scale ranging from “Strongly disagree” to “Strongly agree”, participants rated their acceptance for the following eight domains: perceived ease of use, perceived usefulness, perceived enjoyment, attitude towards food app, social influence, intention to use, system quality, and smartphone experience. In addition, participants were asked to complete two open-ended questions about (i) problems or issues encountered throughout dietary reporting period; and (ii) recommendations for improving the food app. These two questions did not contribute to the acceptability rating.

Besides qualitative analyses, descriptive analyses in the form of frequencies and percentages were used to describe the compliance in dietary reporting (i.e., captured images and text entry). Overall compliance was determined by the frequency of food app use as evidenced in the number of data uploaded to the remote server over the seven-day recording period. In addition, compliance was assessed by how well the participants adhered to instructions to include the fiducial marker in the food images and to provide text description. The five-category ordinal scales for acceptability rating were recoded as “Agree”, “Not sure”, and “Disagree”.

## 3. Results

A total of 28 university students (3 males, 25 females) aged 22.0 ± 0.9 years completed this study. Each participant completed the questionnaire and attended a focus group discussion session after using the food app for seven consecutive days. The majority of the participants were undergraduate students from a Nutrition or Dietetics course (*n* = 16), while the rest enrolled in other health sciences degrees such as Physiotherapy and Biomedical Science programs (*n* = 12). The mean body mass index (BMI) was 20.1 ± 2.3 kg/m^2^ (ranging from 16.4 to 26.0 kg/m^2^).

### 3.1. Compliance in Dietary Reporting

Only 20 (71%) of 28 participants who successfully uploaded their data from the food app to the server were included in the analysis. As shown in Table 1, a total of 673 data entries were uploaded throughout the seven-day food recording period: 194, 242, 183, and 54 were food records for breakfast, lunch, dinner, and snacks, respectively. Out of these uploaded data, 426 were uploaded with food image attachments, while the rest were uploaded without food images (text only). Of the 426 food images uploaded, compliance was good, as 413 (97%) had fiducial markers included in the food images and 378 (89%) had a complete text description for each food image. Only 48 (11%) food images were uploaded without a text description.

**Figure 2:**
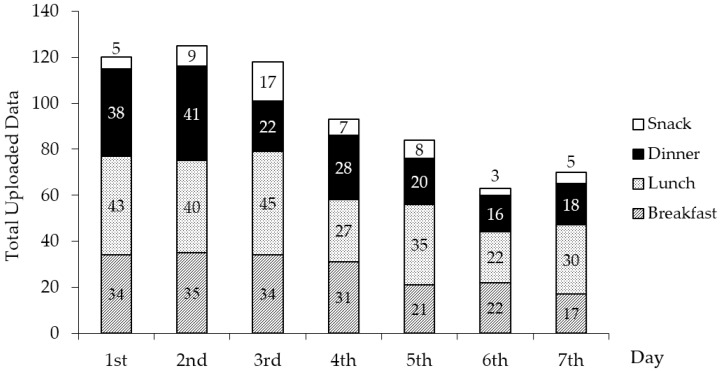
Decreased compliance in 7-day dietary reporting using the food diary mobile application.

On average, dietary intake was recorded for 5.9 days (73% weekdays and 27% weekend days) with an average of 4.8 meal data entries per day for each participant. Eighteen of the 20 participants (90%) reported their dietary intake for at least one meal on three or more days. More than half (55%) of the participants reported at least one meal per day for seven consecutive days. However, the compliance with respect to frequency of food app use reduced from 120 data entries on the first day to 70 data entries on the seventh day. The average decrement compliance was 42% from the beginning towards the end of the seven-day food recording period as shown in Figure 2.

### 3.2. Acceptability in Dietary Reporting

Table 2 shows that participants reported high levels of agreement (agree and strongly agree) in terms of perceived usefulness (69.3%), perceived ease of use (77.1%), attitude (73.6%), perceived enjoyment (62.6%), and smartphone experience (91.1%). Participants also reported moderate levels of agreement regarding quality of the system (40.2%). However, most of the participants were “unsure” and showed moderate levels of agreement in terms of intention to use and social influence.

### 3.3. Focus Group Discussions

Four themes emerged from the analysis of the transcripts: (i) features of the food app; (ii) potential use of the food app; (iii) utility issues; and (iv) suggestions for improvements. The main findings of all the themes are summarized in Table 3.

#### 3.3.1. Theme 1: Features of the Food App

During focus group sessions, participants were asked about the features of the food app that they liked the most. Most of the participants expressed a liking towards the camera or photo-taking function. Many comments were along the lines of “I just want to try to take images” (G2FV), which showed that participants were eager to try photography methods in dietary assessment. Furthermore, participants responded positively about the requirement to provide details of food items in the text. The provision of subsections such as “food name”, “serving size”, and “brand name” displayed in the food app was reported to be useful prompts for them to key in the specific required information into each text box.

#### 3.3.2. Theme 2: Potential Use of the Food App

Participants expressed anticipation of the potential of this image-based food app as a reliable dietary assessment tool. Quotes such as “pictures are more able to show what we have eaten compared to what we jot down using pen…” (G1FW) and “a picture is worth a thousand words” (G2MJ) suggested participants’ positive views towards the food app. Additionally, the food app also enables users to provide dietary intake data in real-time, hence potentially increasing the accuracy of the food data. As one participant stated, “if record using paper-based, users are not recording, instead they recalled, better still to record what they eat on the spot [sic]” (G3FH).

#### 3.3.3. Theme 3: Utility Issues

At the early stages of the dietary recording, participants were keen to provide every detail of the food that they were consuming. However, their motivation subsided gradually over time as one explained, “At the beginning, I key in every detail of the food that I took, day after day, I…” (G5FR). Only “food name” or the main ingredients of a meal were recorded towards the end of the seven-day recording period. Participants perceived that the food app lacked “flexibility” as users were required to enter every detail of their food intake options or copy from previous data entries. Moreover, participants would omit information such as condiments like “spices” and “sauce” in their food recording. In addition, participants encountered difficulties in reporting meals or dishes that were shared in family meals. Participants also reported difficulty in recording snack foods such as “cornflakes” and “tidbits” (G1FQ) especially when they only took one bite to taste or when they share them with friends. For instance: “…like we always eat snack at class, like biscuit and fish crackers, share share [sic]…” (G1FE); “…for the fish crackers, 20 pieces…err…we don’t know how to record [sic]…” (G1FQ).

Of particular concern is that some participants reported changing their food habits during the recording period. “…actually I only eat one complete meal each day, but this week, I ate twice each day…” (G2FN); “I tend to avoid having meal outside…” (G4FC); “I am lazy to record, so I always eat the same food, food that is easy to record” (G1FW) were some of the example of quotes that participants discussed during focus group discussion. Two of the participants also confessed dishonesty in food reporting, “…I will use other foods to replace my food, and actually I didn’t eat that food…” (G4FC). Participants reported that they tend to skip the steps where they were required to key in their leftover food. For example, “…I wouldn’t care whether I have any food leftovers, I will just skip it…” (G2MJ).

Participants regarded the fiducial marker, although thin and easy to carry, as a burden for them to carry along and some tended to forget to place it together with their food when taking food photographs. Participants suggested improving the appearance of the fiducial marker (e.g., non-reflective) to ensure a better image quality. Some technical issues when using the food app were also reported. The common issues include “unable to register into the app”, “cannot save pictures”, “unable to delete”, and “no feedback after syncing”, which affected the intention for continued usage among the participants.

#### 3.3.4. Theme 4: Suggestions for Improvements

Participants saw the need to incorporate more user-friendly features in the application to reduce reporting burden. For instance, allowing “choices” (G2MJ) or “history of recent items” when describing food images, and allowing users to key in their leftover food information at a later time. Other new functions suggested were “barcode scanning” and “voice recognition”.

With regard to fiducial markers, participants suggested one that is easy to carry and “…can stick to the phone, when we want to take photo, sure we will use our phone [sic]…” (G3FH). Other ideas such as “customized reminders”, “rewards”, or “incentives” were suggested to increase compliance among participants in completing their multiple-day dietary records. Participants also requested the provision of a calendar to review their completion progress, time, and date stamps based on the food images taken so that they are conscious of their meal times. An “in-app tutorial” was another suggestion for enabling users to learn how to operate the app when in use, while a “HELP” button would be useful if they encounter difficulties. The design of the application should also be improved in order to attract users’ attention and thus increase their willingness to continue using the food app.

## 4. Discussion

The main aim of this study was to evaluate the acceptability and compliance in reporting dietary intake using an image-based food diary mobile application (food app). Results from this pilot study are important to facilitate on-going adaptation [25] and improvement of the mobile application design as part of the iterative process in product development.

Overall, participants showed high acceptability in terms of perceived usefulness, ease of use, enjoyment, and positive attitude and experience towards using the food app. Participants perceived the application as simple, easy to understand, and easy to use when recording their diets. The results were in accordance with the findings of Wang and colleagues [26] that reported a high level of acceptance among college students towards Wellnavi, a handheld personal digital assistant with camera and mobile telephone card attachment. Adolescents and young adults are typically the earlier and more eager adopters of new technology [14,27], and they have been shown to prefer technology-based dietary assessment methods to traditional paper-based food records [12,28].

While the ratings were high for most domains of acceptability, the intention to use the food app in reporting dietary intake remained low to moderate. This unanticipated finding may be due to the fact that there was a lack of entertaining functions or social networking motivation factors to encourage its use. In a study carried out by Boushey and co-workers [29], an “entertainment oriented” feature appeared as a favorite among adolescents when using the mobile food record (mFR). Therefore, incorporating a gaming component in the food app, such as incentives or rewards, may increase the participants’ motivation to complete their dietary reporting. In line with this, participants also expressed the need to improve the aesthetic and social networking values (appearance, features, functions, interaction, and connection) of the application in order to increase usability. In a study carried out by Aizawa and colleagues [30], there were suggestions to improve the interaction with the application interface in order to increase usability of the application. Another possible explanation for the low intention to use might be that some participants encountered technical issues (e.g., failure to sync their data to the remote server) while using the application. Out of the 28 participants, eight participants failed to sync data saved in their phones with the remote server. This was likely caused by the low specification phones used by the participants (e.g., Samsung Galaxy Grand Prime). The limited resources such as low Random Access Memory (RAM) capacity in these phones caused the app to crash, which resulted in data loss within the SQLite database. This may have hampered the participants’ willingness and motivation to continue using the food app for a week. Taken together, these findings supported the suggestion to incorporate some aspects of gaming and improvements in user experience to strengthen the intention to use the food app.

With respect to compliance in reporting, participants completed dietary intake record for 5.9 days with an average of 4.8 occasions of meal data being uploaded per day. Out of the total 426 food images captured, 89% were considered complete in terms of provision of food images and text descriptions. It is encouraging to compare this relatively high figure with that described by Casperson and co-workers [14] who reported that only an average of 2.2 eating events were recorded daily over a 3-day period using a mobile phone food record app (FRapp) in a sample of young adolescents aged 11–14 years. Nonetheless, previous studies have suggested that girls were more likely than boys [29], and adults were more likely than adolescents [13] to capture images of their meals. Therefore, with a sample comprising mostly females (89%), we are conscious of the possible gender and age bias that may have resulted in higher reporting compliance in this study.

Another way of evaluating the compliance was to observe whether participants included the credit card-sized fiducial markers in their food images. The size of a fiducial marker is important in deciding its amenability, as a marker that is either too large or small would discourage its use [2,13]. One study reported that 61% of 20 participants adhered to the requirement of including a fiducial marker in their food images [14]; while another study showed that 69% from a sample of 78 adolescent subjects and 79% from 56 adult subjects included fiducial markers in their food images [13]. In the study carried out by Six and colleagues [2], which collected data for two meal occasions, only a small proportion of the subjects included fiducial markers in both the pre- and post-meal images of their foods for the first meal (71%, 50/70) and slightly more for the second meal (77%, 54/70). Our study reported that 97% of participants included the fiducial marker provided into every food image, showing high cooperation among participants in adhering to the protocol. Some of the participants found it easy to carry a thin credit card size fiducial marker along with their university identification card. Nevertheless, some participants reported that they occasionally forgot to include the fiducial markers while capturing a picture of their meals. Another concern was that the reflection from the glossy surface of the fiducial marker had affected their photos quality. To counter these problems, some participants suggested having an in-built or electronic fiducial marker, which could alert the users when the fiducial marker was not visible. These suggestions were consistent with those put forward in previous studies [14,29].

In the focus group discussion sessions, some participants reported that they tended to eat more meals in order to complete recording in the food app, while some replaced their typical meals with other simpler ones to ease recording. This reactivity might be promoted by self-consciousness of being judged by a third party. Furthermore, a decreasing trend of food data provision towards the end of food recording was observed. The overall rate of decrease was 42% from the first day towards the seventh-day of food recording period (see Figure 2). This reduced compliance over time is expected due to respondent fatigue imposed by the tedious requirements of keeping a multiple-day food record. In addition to taking food photographs, participants were also asked to fill in detailed textual food information over seven consecutive days. Consequently, some participants lost motivation in providing full description of their food intake (as reported in focus group discussion) while some logged less food records, which reduced the reliability of the records over time. This finding is in accordance with the observation of Rebro and colleagues [31], which reported that users reduced the quality of food recording after keeping records for more than four days. While it has been suggested that an image-based food record is more effective in generating more food records than a text-based record [30], it remains unknown whether omitting textual data (to reduce respondent burden) could provide satisfactory reporting without compromising data quality. Future studies may need to provide strategies or alternatives in addressing this respondent fatigue issue.

Among suggestions to improve the existing food app was to incorporate user training and action reminders in the application. In this pilot study, the user training was minimal and was supplemented by a written manual. Participants reported the need to have in-app tutorial and to provide a “HELP” button as well as a search engine in order to guide and increase the compliance rate among participants. Although reminders to prompt users to undertake certain actions are useful [29,32,33], this feature should be carefully included as users may feel irritated at receiving numerous alerts and some may become demotivated at completing the required actions [34]. Therefore, all the reminders should be customizable to individuals according to different situations.

There are some limitations in this study. First, nutrient intake was not calculated from the food records; therefore, misreporting (under/over-reporting) could not be determined. Nutrient analysis could not be accomplished, as the development of an in-house nutrient database was incomplete during this pilot study. Second, this study used a modified questionnaire to rate acceptability; however, the validity of the eight domains to predict the ultimate use of the food app remains unknown for the Malaysian population. Third, this study only included university undergraduate students from the health sciences background and who were presumably also highly literate in information technology. Therefore, these participants may be highly motivated and subject to reporting bias in the direction of increased acceptability. This limitation in sample selection may compromise generalizability of the study findings to the broader adult population. Moreover, the food app is developed on the Android platform only. Therefore, feedback could not be obtained from smartphone users of other operating systems.

This is the first study in Malaysia that documents the feasibility of reporting dietary intake using a newly developed image-based food diary mobile application. The main strength of this study lies in the use of both quantitative and qualitative methods in verifying the acceptability and compliance in dietary reporting. Apart from using questionnaires, follow-up focus group discussions were employed to allow in-depth understanding of the users’ perceptions, preferences, and opinions towards the food app. In addition, this study employed a consecutive seven-day recording protocol to document the changes in dietary reporting over an extended period.

## 5. Conclusions

The findings from this pilot study suggested that the Android-based food diary mobile application has great potential in photographing food images and collecting dietary data. Young adults showed good acceptability and compliance in using the mobile application to record their diets. Forming part of the evidence-based development of a dietary assessment tool, the next step of this work includes improvement of the food app prototype based on feedback from the participants. Further evaluation including validation in different age groups is required to establish the food diary mobile application as a valid dietary assessment tool.

## Figures and Tables

**Table 1:** Compliance in dietary reporting.

Criteria	Frequency, *n*	Percentage, %
Total data uploaded ^1^	673	
Breakfast	194	29
Lunch	242	36
Dinner	183	27
Snacks	54	8
Total food image recorded ^2^	426	
with fiducial marker	413	97
with text description ^3^	378	89
without text description	48	11
Average number of recording day (days)	5.9	
Weekdays		73
Weekends		27
Average number of uploaded data per day	4.8	

^1^ Including data in different formats (image, text, combination of image and text) uploaded from the food app to the remote server; ^2^ Refers to pairs of images taken at 90 degree and 45 degree angles; ^3^ Refers to text information on food name, serving size, preparation method, brand name, and other additional food information.

**Table 2:** Level of acceptability towards the food app using the modified TAM ^1^ questionnaire (*n* = 28).

Domains	Agree and Strongly Agree (%)	Disagree and Strongly Disagree (%)	Not Sure (%)
Perceived Usefulness	69.3	7.8	22.9
Perceived Ease of Use	77.1	12.1	10.7
Attitude	73.6	4.3	22.1
Intention to Use	38.1	23.8	38.1
System Quality	40.2	32.1	27.7
Social Influence	33.4	20.2	46.4
Perceived Enjoyment	62.6	10.7	26.8
Smartphone Experience	91.1	5.3	3.6

^1^ Technology Acceptance Model (TAM).

**Table 3:** Summary of findings from the focus group discussions.

Themes	Summary of Findings
Theme 1: Features of the food app	Preferred photo-taking or camera function. Good prompts provided to fill in required textual information.
Theme 2: Potential use of the food app	App with in-built camera function will provide more details regarding food consumed.
Theme 3: Utility issues	Decreased compliance rate overtime. Dishonesty in reporting food intake. Technical issues such as failure in installing the application, saving images, and syncing data.
Theme 4: Suggestions for improvements	Provide more user-friendly features and guidance. Provide new functions such as barcode scanning, in-app tutorial. Other additional features to increase compliance rate such as customized reminders, incentive-based component. Increase the aesthetic value of the food app.
